# Transcriptome Analysis of Tetraploid and Octoploid Common Reed (*Phragmites australis*)

**DOI:** 10.3389/fpls.2021.653183

**Published:** 2021-05-05

**Authors:** Cui Wang, Tong Wang, Meiqi Yin, Franziska Eller, Lele Liu, Hans Brix, Weihua Guo

**Affiliations:** ^1^Institute of Ecology and Biodiversity, Shandong Provincial Engineering and Technology Research Center for Vegetation Ecology, School of Life Sciences, Shandong University, Qingdao, China; ^2^Organismal and Evolutionary Biology Research Program, Faculty of Biological and Environmental Sciences, University of Helsinki, Helsinki, Finland; ^3^College of Landscape Architecture and Forestry, Qingdao Agricultural University, Qingdao, China; ^4^Department of Biology, Aarhus University, Aarhus, Denmark

**Keywords:** *Phragmites*, transcriptomics, polyploid, stress tolerance, evolution

## Abstract

Polyploidization in plants is thought to have occurred as coping mechanism with environmental stresses. Polyploidization-driven adaptation is often achieved through interplay of gene networks involved in differentially expressed genes, which triggers the plant to evolve special phenotypic traits for survival. *Phragmites australis* is a cosmopolitan species with highly variable phenotypic traits and high adaptation capacity to various habitats. The species’ ploidy level varies from 3x to 12x, thus it is an ideal organism to investigate the molecular evolution of polyploidy and gene regulation mediated by different numbers of chromosome copies. In this study, we used high-throughput RNAseq data as a tool, to analyze the gene expression profiles in tetraploid and octoploid *P. australis*. The estimated divergence time between tetraploid and octoploid *P. australis* was dated to the border between Pliocene and Pleistocene. This study identified 439 up- and 956 down-regulated transcripts in tetraploids compared to octoploids. Gene ontology and pathway analysis revealed that tetraploids tended to express genes responsible for reproduction and seed germination to complete the reproduction cycle early, and expressed genes related to defense against UV-B light and fungi, whereas octoploids expressed mainly genes related to thermotolerance. Most differentially expressed genes were enriched in chaperones, folding catalysts and protein processing in endoplasmic reticulum pathways. Multiple biased isoform usage of the same gene was detected in differentially expressed genes, and the ones upregulated in octoploids were related to reduced DNA methylation. Our study provides new insights into the role of polyploidization on environmental responses and potential stress tolerance in grass species.

## Introduction

Polyploidization is an important evolutionary force for shaping genetic diversity in eukaryotes (Stebbins, 1950). Polyploidizations can result in the emergence of new lineages within species, working as a driver of intraspecific diversification or even resulting in speciation. Chromosome doubling can promote novel phenotypic traits, and has therefore been proposed to greatly increase species diversification (Crow and Wagner, 2005; Landis et al., 2018). About 70 percent of all angiosperms arose from chromosome doubling, among which nearly all Poaceae species originated from the same diploid ancestor (Masterson, 1994; Salse et al., 2008; del Pozo and Ramirez-Parra, 2015). Polyploidization, including allopolyploidization, autopolyploidization, and segmental polyploidization, is often seen among closely related plant species, and multiple polyploidization events can occur within the same species of certain genera, such as *Inga, Senna, Leucanthemum*, and *Dupontia* (Brysting et al., 2004; Figueiredo et al., 2014; Cordeiro and Felix, 2018; Wagner et al., 2019). Compared to diploids, polyploids usually have larger stomata and leaf area, increased pollen-grain size, and higher germinal pore numbers (Tamayo-Ordóñez et al., 2016; Liqin et al., 2019). These traits are considered to be advantageous in unfavorable environments, thus polyploids are often more tolerant to environmental stresses, such as drought, salinity, cold, heat, or nutrient deficiency (del Pozo and Ramirez-Parra, 2015). In addition, the evolution of polyploids is often coupled with asexual reproduction, such as apomixis (Schinkel et al., 2016; Hojsgaard and Hörandl, 2019), vegetative propagation and perennial growth, facilitating clonal spreading and increased survival rates under extreme conditions. Species featuring those traits can be highly adaptive to novel environments, and in some cases even become invasive (Te Beest et al., 2012).

Due to the high number of allelic copies, polyploids may develop unique gene expression systems to coordinate the function of multiple genomic copies, and balance the interaction between homeologs in allopolyploids (Yoo et al., 2013). Comparison of gene expression in allopolyploid and diploid *Populus* species revealed considerable differences between gene expression among different ploidy levels, resulting in overall superior phenotypic traits in polyploids. Differential expression of protein kinase genes, growth-regulating factors and hormone-related genes were largely responsible for the development of those phenotypic differences (Liqin et al., 2019). Those genes are also involved in stress-activated pathways and, hence, initiate adaptive responses to stress signaling in plant development (Golldack et al., 2014). Therefore, investigating genes expressed as a function of ploidy level is important to understand what advantages polyploidization has for plant evolution.

*Phragmites australis* is a cosmopolitan grass species with high intraspecific variability of ploidy levels, including 3x, 4x, 6x, 7x, 8x, 11x, 12x, x = 12 (Gorenflot, 1986). The most common seen cytology for *P. australis* in nature is tetraploid and octoploid. Tetraploids are distributed over most of the temperate region, and octoploids are found to occur mainly in South Africa, Romania, Greece and East Asia (Connor et al., 1998). *Phragmites australis* is able to tolerate extreme environmental conditions, and its suitable habitats include freshwater ponds, saline coastlines, dunes with severe aridity, and oligo- to polyhaline salt meadows (Wen-Ju et al., 1999; Song et al., 2020). Previous studies have proposed that different ploidy levels do not cause phenotypic changes (Achenbach et al., 2012) or higher tolerance to salinity (Achenbach et al., 2013). In contrast, it has been found that octoploid *P. australis* were less affected by salt stress than tetraploids (Paucã-Comãnescu et al., 1999), while a recent finding showed the European lineage haplotype O (which is mainly tetraploid) was likely to be more tolerant to soil salinity than East Asian clades of haplotype P, which are more frequently octoploids (Lambertini et al., 2020; Liu et al., 2020). Ploidy has been emphasized as a key factor affecting the adaptation to new territories, for example allowing European tetraploid lineages to spread to Asian habitat (Lambertini et al., 2020), and enabling their invasion in North American environments (Pyšek et al., 2018). Despite those apparent advantages of a tetraploid genome, a large genome size may also be advantageous for certain traits of *P. australis* (Suda et al., 2015; Meyerson et al., 2016). Thus, octoploid *P. australis* have lower aphid colonization, bigger leaves, thicker shoots and taller, sturdier stems than tetraploids (Hanganu et al., 1999; Hansen et al., 2007; Lambertini et al., 2012; Meyerson et al., 2016; Eller et al., 2017). However, there is no systematic study to date investigating, how ploidy level affects gene expression of *P. australis*, which could demonstrate if underlying mechanisms determined by polyploidy control phenotypic traits.

In this study, we used transcriptomics on octoploid and tetraploid *Phragmites* individuals from a common garden to unravel potential intraspecific differences in gene expression profiles. Our aim was to understand how polyploidy affected the transcriptome in different *P. australis* genotypes grown in the same environment.

## Methods

### Sampling

Leaf samples of six individuals were selected for transcriptome analysis, comprising three individuals of octoploids, and three individuals of tetraploids (Table 1). At least 10 healthy young leaves were collected from each individual from a common garden (Coordinates: 36.43°N, 117.43°E) at Shandong University in July, 2020. The leaves were immediately submerged into RNA-sample-preservation solution (Coolaber, Beijing, China), which keeps the RNA intact and protected from degradation. The leaf samples were then stored at 4°C in a fridge overnight, and sent to Shanghai Honsun bio Company^1^ for RNA extraction and next generation sequencing. Total RNA isolated from each replicate was sequenced using the Illumina Hiseq Xten platform. The ploidy level of each plant was confirmed by flow cytometry, following the protocol in Meyerson et al. (2016). The resulting sequences were deposited in the NCBI Sequence Read Archive (SRA) database with the following identifiers: BioProject PRJNA687616.

**TABLE 1:** Sample information of the RNA-seq data used in this study.

Species name	Sample name	Mapping rate	Number of reads (million)	Ploidy level	Origin	Coodinates	Code used in other studies
*Phragmites australis*	S136-1	83.63%	67.87	8	Australia	34°56′00.0″S 138°36′00.0″E	FEAU136
*Phragmites australis*	S150-1	84.54%	59.15	8	Australia	34°28′00.0″S 146°01′00.0″E	FEAU150
*Phragmites australis*	S162-1	83.98%	72.28	8	Australia	36°09′00.0″S 147°00′00.0″E	FEAU162
*Phragmites australis*	S191-1	84.43%	62.84	4	United States	43°16′35.0″N 77°16′40.0″W	NAint191
*Phragmites australis*	S207-1	84.37%	66.80	4	Italy	45°41′00.0″N 9°46′00.0″E	EU207IT
*Phragmites australis*	S620-1	80.09%	62.86	4	Czech Republic	48°39′00.0″N 14°22′00.0″E	EU620

### Genome Assembly and Annotation

To facilitate the genomic mapping of transcriptomic reads, we assembled and annotated the genome of *P. australis* using next generation sequencing (NGS) reads produced by BGISEQ-500 sequencer. Whole genome sequences were obtained from NCBI SRA database (Accession: SRX4043155) (Liu et al., 2019). The genome was assembled with MaSuRCA 3.3.3 assembler using default settings (Zimin et al., 2013). The draft genome assembly was curated with Purge Haplotigs v1.0.4 to remove wrongly assembled contigs which are heterozygous to the real reference (Roach et al., 2018). Gene prediction was performed by both homology based and *ab initio* methods. Genome assembly and annotation with *Miscanthus sinensis* were obtained from Phytozome^2^ to serve as a reference for homology prediction using GeMoMa v1.6.4 (Keilwagen et al., 2019). *Ab initio* gene prediction was performed using GeneMark-ES v4.64 (Lomsadze et al., 2005), BRAKER2 v2.1.5 (Brůna et al., 2021) and PASA v2.4.1 (Haas et al., 2011). RNAseq data of one octoploid individual was aligned to the draft assembly by STAR aligner v2.7.6a (Dobin et al., 2013), and used as evidence to define intron borders in BRAKER2 prediction. *De novo* assembly of RNAseq data was performed using TRINITY v2.12.0 (Grabherr et al., 2011) and used in PASA to get a high quality dataset for *ab initio* gene predictions. We integrated all evidence of gene prediction in EvidenceModeler v1.1.1 (Haas et al., 2008) to get the consensus gene structure.

### Transcriptome Assemblies

To date the divergence time between the octoploid and tetraploid, we included data of *Zea mays*, *Arundo donax*, and *Phragmites karka* to provide calibration points. RNA-seq from leaf tissue of four individuals of *P. karka* (Accession Nos.: SRR9670021, SRR9670022, SRR9670025, and SRR9670026) and one individual of *A. donax* (Accession No.: SRR8083515) were obtained from NCBI biosample database from previous studies (Evangelistella et al., 2017; Nayak et al., 2020). Transcriptome assembly of *Z. mays* was downloaded from Transcriptome Shotgun Assemblies in NCBI (Table 1). Transcripts of *P. australis* octoploids and tetraploids were assembled using TRINITY v2.12.0 (Grabherr et al., 2011) separately in genome-guided *de novo* mode, and RNA-seq data of *P. karka* and *A. donax* were assembled in *de novo* mode using TRINITY v2.12.0. The Open Reading Frame (ORF) of each transcriptome assembly was predicted using TransDecoder v5.5.0 (Haas et al., 2013), and the recognized protein coding sequences were used to infer orthogroups and orthologs in OrthoFinder v2.4.0 (Emms and Kelly, 2019). Both orthologs and paralogs are homologs among species, and they differ in the way that orthologs were directly descendent from the most recent common ancestor and are results of speciation, whereas paralogs within species were created from duplication of the orthologs and are often results of Whole Genome Duplication (WGD). By integrating several programs in the pipeline, OrthoFinder first inferred a rooted species tree based on the clustering the gene trees of input amino acid sequences, and then inferred orthogroups among species.

### Divergence Time Between Ploidy Levels

Compared to paralogues, orthologues are the genomic regions that are directly transmitted from the most common ancestor without genomic duplications and reallocation, thus orthologues reflect the true phylogeny. We aligned 98 single copy orthologue sequences in all species using MAFFT v7.429 (Katoh et al., 2002), and calculated the divergence time of each node using BEAST2 v2.6.1 (Bouckaert et al., 2014) with a strict clock model and a Blosum62 + G (four rate categories) site model. Previous studies estimated the Most Recent Common Ancestor (MRCA) of the PACMAD clade including *Z. mays* and *P. australis* to be at 44 Million Years Ago (MYA) (Vicentini et al., 2008), so we set the parameter of TMRCA as log normal distribution, with the Mean in Real Space checked, an offset of 40.0 MY, a mean of 6.0 MY and a standard deviation of 0.5 MY. The chain length of the Markov Chain Monte Carlo was set to ten million, with sampling every 5,000 states. Tracer v1.7.1 was used to estimate the convergence of the run, and a convergence state was considered to be reached if the effective sample size (ESS) of all parameters was at least 200.

### Read Mapping

RNA sequencing produced between 59.15 and 72.28 million 2 × 150b pair-end reads for each sample in this study. RNA-seq reads of each sample were cleaned and mapped on to the genome assembly to obtain the read count of each gene. Quality of the RNA-seq reads was checked with FastQC v0.11.8 (Andrew, 2010). Only reads with Phred score higher than 30 were kept, and overrepresented sequences were removed from the library using cutadapt 2.7 (Martin, 2011). The clean reads were aligned to *P. australis* draft genome using STAR aligner 2.7.1a two pass procedure (Dobin et al., 2013), and the bam files were sorted with samtools 1.10 (Li et al., 2009). Transcriptome abundance estimates were performed with StringTie v2.1.4 (Pertea et al., 2015). All transcripts were then merged and assembled to a consensus transcript set. We aligned RNAseq data of each sample to the merged transcript using command (stringtie -e -B). The resulting coverage data were later transformed to gene count matrix by stringtie script prepDE.py.

### Differential Gene Expression Across Ploidy Levels

To find out the genes that are differentially expressed between groups, rather than within group, we analyzed the read counts of each gene using R package DEseq2 v1.30.1 (Love et al., 2014) in the R environment v3.6.1. After internal normalization, DEseq2 calculate size factor for each gene in each sample to correct for library size, and uses shrinkage estimation to estimate dispersions and fold changes among biological replicates, and then fits negative binomial generalized linear models for each gene and uses the Wald test for significance testing (Love et al., 2014). Genes showing absolute values of a log_2_ fold-change (LFC) higher than 2 were considered as differentially expressed gene (DEG). The adjusted *P*-value was adopted to control for the false discovery rate due to multiple testing using the Benjamini and Hochberg methods in DEseq2, and a *P*-value lower than 0.001 was regarded to be statistically significant. The top 500 genes with highest row variance were selected to perform a Principal Component Analysis (PCA) to assess the effects of external variation on gene expression.

### Functional Annotation of the Genome and Novel Genes

To characterize the molecular functions of the DEGs, we first blasted the genome against available protein databases to get functional annotation of each gene, and then searched the DEGs against the genome to subtract the corresponding functions. Protein function of the annotated genome was estimated through Mercator4 V2.0 (Schwacke et al., 2019). Transcription factors were predicted from the online tool plantTFDB v5.0 (Jin et al., 2016). Amino acid sequences of novel transcripts produced by StringTie were extracted using IsoformSwitchAnalyzeR v1.12.0 (Vitting-Seerup and Sandelin, 2019), and searched against pfam-A protein database using Pfamscan to obtain the domain information (Mistry et al., 2007). The novel transcripts sequences were also annotated from eggNOG-mapper v2 to get a more complete information of the genes (Huerta-Cepas et al., 2017). Annotation information including GOslim and gene association files of *Arapdopsis thaliana* was downloaded from The Arabidopsis Information Resource^3^. The reference genome was first aligned to *A. thaliana* using BLASTp algorithms (*e*-value < 10^–5^), and then to map the genes to *A. thaliana* to obtain the gene ontology (GO) terms clustered based on biological process, cellular component or molecular function. GO term enrichment analysis was conducted with GOAtools v1.0.15 using Bonferroni correction with a cut-off threshold of *P* < 0.01 (Klopfenstein et al., 2018). KEGG analysis was performed through KAAS server and gene enrichment was done in R package “clusterProfiler” v 3.18.1 (Yu et al., 2012).

### Alternative Splicing

To detect whether alternative splicing has played a role in gene regulation of different ploidy levels, we performed a test on the transcriptomes using IsoformSwitchAnalyzeR (Vitting-Seerup and Sandelin, 2019). Isoform switches were predicted using DEXSeq v1.36.0 (Anders et al., 2012), with parameter set to alpha = 0.05, dIFcutoff = 0.1, in which case isoforms were only considered to be switching when there was more than 10% of the changed isoforms. Genome wide alternate splicing and potential functional consequences of the identified isoform switches between the tetraploid and octoploid sets, especially the isoforms in differentially expressed genes were predicted.

## Results

### Genome Assembly and Functional Annotation

**FIGURE 1:**
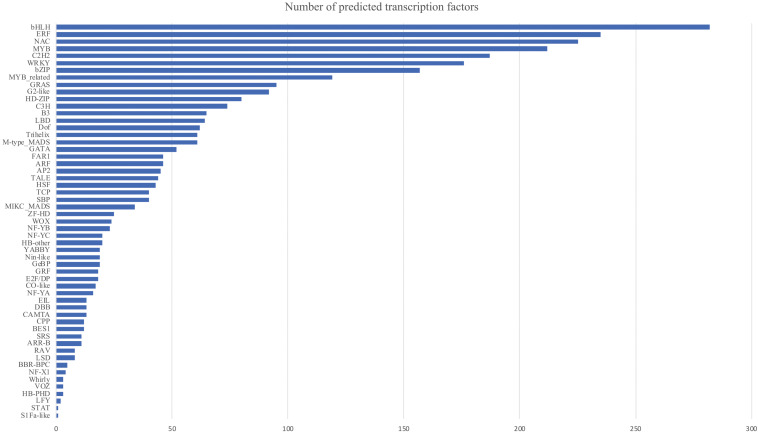
Number of predicted transcription factors in *Phragmites australis* genome. The transcription factors were obtained by searching the annotated reference genome against the Plant TFDB V5.0. Genes annotated to the same transcription factor families were counted as one class. Details of transcription factor names can be retrieved from http://planttfdb.gao-lab.org/.

The genome size of *P. australis* was about 912.58 Mb, with heterozygosity of 1.31%. The N50 contig length of the new assembly was 36,770 bp. In total, 141,683 genes were annotated in the draft genome. The annotated genome was classified into 28 functional categories by Mercator4, with genes distributed in 67–100% of Mercater4 leaf bins. Among all genes in the draft genomes, 15.48% were annotated with Mercator4, and the number of genes in each top bin varied from 0.03 to 2.72% (Table 2). A total of 2,998 transcription factors (TFs) were predicted, specifying 55 types, and the most identified TFs (>100 genes) included bHLH (282 genes), ERF (235 genes), NAC (225 genes), MYB (212 genes), C2H2 (187 genes), WRKY (176 genes), bZIP (157 genes), and MYB related (119 genes) (Figure 1).

**TABLE 2:** Gene annotation inferred by Mercator4.

Top level bins classifying biological process	Number of leaf bins	*P. australis* occupied leaf bins	*P. australis* number of genes	Percent of the total genes (%)	Upregulate (gene number)	Downregulate (gene number)
1 Photosynthesis	230	172	404	0.285	0	0
2 Cellular respiration	130	107	303	0.214	0	0
3 Carbohydrate metabolism	110	106	376	0.265	0	0
4 Amino acid metabolism	134	127	336	0.237	0	0
5 Lipid metabolism	191	178	732	0.517	0	0
6 Nucleotide metabolism	58	58	149	0.105	0	0
7 Coenzyme metabolism	161	154	325	0.229	0	0
8 Polyamine metabolism	15	13	37	0.026	0	1
9 Secondary metabolism	100	67	202	0.143	0	0
10 Redox homeostasis	48	46	192	0.136	0	0
11 Phytohormone action	147	133	842	0.594	1	1
12 Chromatin organization	142	133	471	0.332	0	0
13 Cell cycle organization	274	264	710	0.501	1	2
14 DNA damage response	82	81	131	0.092	0	0
15 RNA biosynthesis	285	273	3,859	2.724	3	4
16 RNA processing	358	329	844	0.596	0	1
17 Protein biosynthesis	396	358	972	0.686	0	0
18 Protein modification	291	286	2,015	1.422	1	2
19 Protein homeostasis	289	283	1,665	1.175	0	3
20 Cytoskeleton	118	110	494	0.349	0	1
21 Cell wall	135	123	848	0.599	2	0
22 Vesicle trafficking	192	195	736	0.519	0	0
23 Protein translocation	141	135	325	0.229	0	0
24 Solute transport	174	171	1,860	1.313	0	3
25 Nutrient uptake	56	47	222	0.157	0	0
26 External stimuli response	116	101	357	0.252	1	0
27 Multi-process regulation	74	72	417	0.294	0	0
50 Enzyme classification	50	39	2,108	1.488	4	8

### Map Efficiency of RNAseq Data and Differential Gene Expression

**FIGURE 2:**
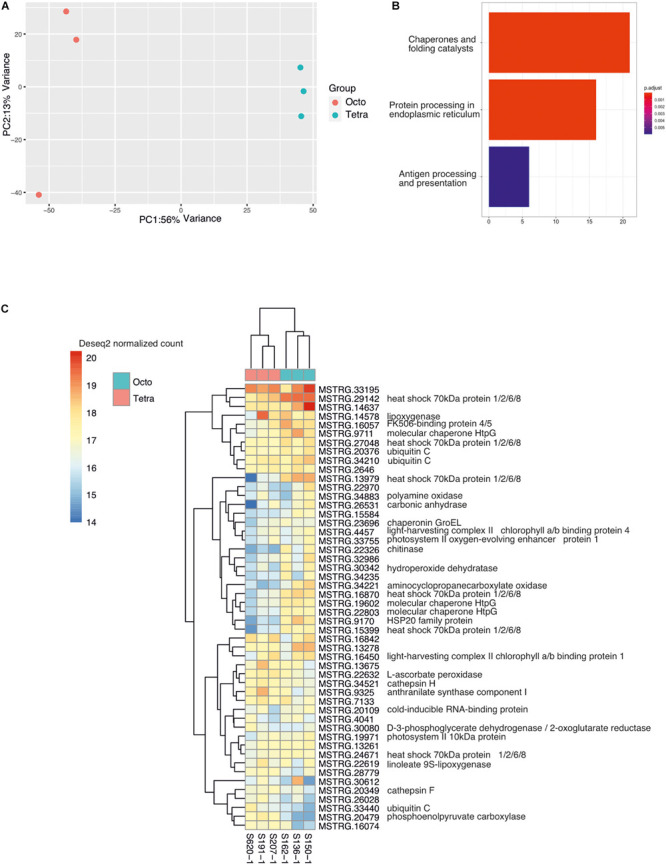
Data visualization and differential gene expression of the transcripts between octoploid and tetraploid *Phragmites australis*. **(A)** Principal Component Analysis (PCA) of the transcript count transformed with rlog function from all samples. **(B)** Enriched Kyoto Encyclopedia of Genes and Genomes (KEGG) pathway of the genes that are upregulated in octoploids. The horizontal axis indicates number of genes. **(C)** Hierarchical clustering of genes with the highest mean of normalized counts across all samples. Abbreviated gene names are followed by a functional annotation of that gene.

All samples have high percentage (>80.09%) of RNAseq data mapped on the genome draft assembly (Table 1). PCA showed that the first two components explain 69% of the variance, of which most of the variation (56%) was explained by PC1, which separated the samples into tetraploid and octoploid groups (Figure 2A). Of the 49,024 genes expressed in both octoploids and tetraploids, the expression level of 1,395 transcripts were significantly different between the two ploidy levels (Wald test, *P* < 0.01). There were 439 transcripts upregulated and 956 transcripts downregulated in tetraploids compared to octoploids (Wald test, *P* < 0.01). Altogether, protein domains of 427 out of the 439 (97.27%) upregulated transcripts and 879 out of 956 (91.95%) downregulated transcripts were annotated from the pfam-A database.

### Function Enrichment of DEG

Using Mercator4 annotation, the upregulated genes were identified to be related to RNA biosynthesis, cell wall, phytohormone action, cell cycle organization, protein modification, and external stimuli response (Table 2). The downregulated genes were found to be involved in the biological processes including RNA biosynthesis, protein homeostasis, solute transport, cell cycle organization, protein modification, polyamine metabolism, phytohormone action, RNA processing, cytoskeleton (Table 2). KEGG pathway enrichment suggested downregulated genes were significantly related to “Chaperones and folding catalysts,” “Protein processing in endoplasmic reticulum,” and “Antigen processing and presentation” pathways (Figure 2B). We did not find KEGG enrichment with the upregulated genes. GO enrichment analysis assigned 174 GO terms to the upregulated genes, among which we found 24 cell components (CC), 61 molecular functions (MF), and 89 biological processes (BP) (Supplementary Table 1). Biological processes were mainly metabolic processes (26 GO terms), responses to stimuli (5 GO terms), responses to stress (4 GO terms), but also involved in development, reproduction and seed germination. We assigned 211 GO terms to the downregulated genes, including 28 cell components, 63 molecular function, and 120 biological process (Supplementary Table 2). Downregulated genes were mostly enriched in biological processes such as metabolic processes (26 GO terms), protein folding and responses to unfolded or incorrectly folded proteins (9 GO terms), responses to stimuli (9 GO terms), responses to stress (9 GO terms), telomere maintenance (4 GO terms), and response to heat stress (3 GO terms). Genes with the highest mean of normalized counts across all samples showed the most abundant expressed genes are heat shock proteins (HSP70, HSP20), chaperone (HtpG), and ubiquitin C (Figure 2C). Seven types of transcription factors were found in upregulated genes, including Nin-like (2), NAC (1), B3 (1), ERF (3), HY5 (1), bZIP (1) and ARR-B (1). Ten types of transcription factors were identified in downregulated genes, including bHLH (1), SBP (7), bZIP (11), ERF (2), HB-other (3), MYB-related (2), BBX-DBB (1), GATA (1), GARP (1) and WRKY (1) (Table 3).

**TABLE 3:** Transcription factors identified in differentially expressed genes.

Upregulated		Downregulated	
Transcript ID	Transcription factor	Transcript ID	Transcription factor
MSTRG.20270.1	Nin-like	MSTRG.1104.2	bHLH
MSTRG.20270.2	Nin-like	MSTRG.15405.1	SBP
MSTRG.4654.1	NAC	MSTRG.15405.3	SBP
MSTRG.25162.1	B3	MSTRG.15405.5	SBP
MSTRG.5559.1	ERF	MSTRG.15405.6	SBP
MSTRG.5559.2	ERF	MSTRG.15405.7	SBP
MSTRG.5559.3	ERF	MSTRG.15405.8	SBP
EVMevm.TU.jcf7180004141171.8	ARR-B	MSTRG.15405.9	SBP
EVMevm.TU.jcf7180004129794.10	HY5	MSTRG.1548.1	bZIP
evm.model.jcf7180004129794.10	bZIP	MSTRG.1548.2	bZIP
		MSTRG.1548.3	bZIP
		MSTRG.24846.1	bZIP
		MSTRG.24846.2	bZIP
		MSTRG.24846.3	bZIP
		MSTRG.27569.1	bZIP
		MSTRG.27569.2	bZIP
		MSTRG.27569.3	bZIP
		MSTRG.27569.4	bZIP
		MSTRG.27569.5	bZIP
		MSTRG.24613.1	ERF
		MSTRG.24613.2	ERF
		MSTRG.22481.1	HB-other
		MSTRG.22481.4	HB-other
		MSTRG.22481.5	HB-other
		MSTRG.22330.1	MYB_related
		MSTRG.22330.3	MYB_related
		EVMevm.TU.jcf7180004099680.9	BBX-DBB
		EVMevm.TU.jcf7180004112813.1	GATA
		EVMevm.TU.jcf7180004088796.12	GARP
		EVMevm.TU.jcf7180004037963.3	WRKY

### Alternative Splicing

**FIGURE 3:**
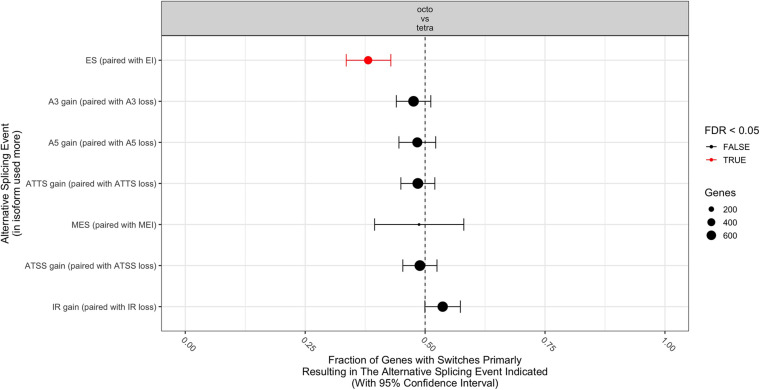
Compared consequences of alternative splicing event between ploidy levels (octo- and tetraploid) in *Phragmites australis*, inferred from biased isoforms in each group. Fraction of genes with switches primarily resulting in the alternative splicing event were indicated with 95% Confidence Interval. Data labeled with red indicated the significant trend, with False Discovery Rate < 0.05. Significant isoform usage was indicated with an asterisk. *p-value < 0.05, ***p-value < 0.001, ns, no significant difference. Compare to octoploids, tetraploids showed higher percentage of genes (about 72%) with consequences of Exon Inclusion (EI), and only around 38% of the genes showed consequences of Exon Skipping (ES).

**FIGURE 4:**
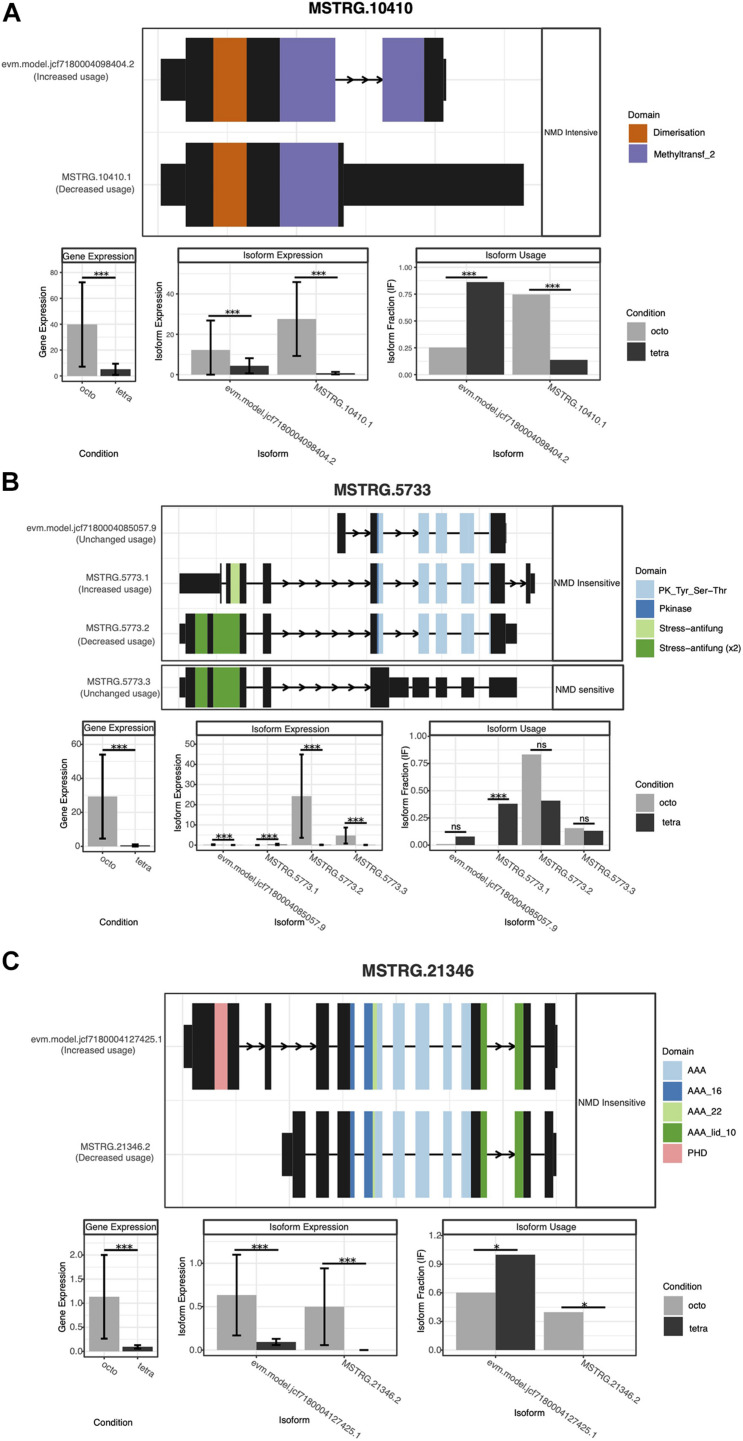
Structural and expression analysis of gene MSTRG.10410 **(A)**, MSTRG.5733 **(B)** and MSTRG.21346 **(C)** for which with alternative splicing events has biological consequences. Isoforms insensitive and sensitive to Nonsense Mediated RNA Decay (NMD). *p-value < 0.05, ***p-value < 0.001, ns, no significant difference. For each gene, the top graph displays gene structure of the isoform, with domains annotated from Pfam database indicated. The bar graph on the left showed the differential gene expression, and the bar graph in the middle indicated the differential isoform expression, and the bar graph on the right represented isoform usage bias between octoploids and tetraploids.

In total, 1,596 genes showed at least one isoform. Among these genes, 2,554 isoforms and 2,417 switches were identified. With the dIF cutoff threshold set as 0.1, we analyzed the consequences of 1,282 genes that had 2,049 isoforms, with 2,024 switches. Compared to octoploids, tetraploids showed significant biased usage of Exon Inclusion (EI) than Exon Skipping (ES) (Figure 3). Octoploids expressed a slightly higher level of ES than tetraploids (Supplementary Figure 1). Among the genes that were upregulated in tetraploids relative to octoploids, 19 genes showed a significant biased use of isoforms (*p* < 0.05), and among the genes that were downregulated in tetraploids, 31 genes were found to code for significantly biased isoforms (Table 4 and Figure 4). Premature termination codons (PTCs) were frequently found in repeat regions, such as gene “MSTRG.3765” (10 isoforms) and “MSTRG.6510” (5 isoforms) in family PRR and WD40. Most of the biased usage of isoforms were Nonsense Mediated RNA Decay (NMD) insensitive, but a few isoforms were NMD sensitive (Table 4 and Figure 4).

**TABLE 4:** Biased isoform switches in differentially expressed genes.

Condition 1	Condition 2	Upregulated			Downregulated		
		Isoform ID	Domain changed	NMD sensitivity	Isoform ID	Domain changed	NMD sensitivity
Octoploid	Tetraploid	MSTRG.31526.1, MSTRG.31526.2	DIOX_N	Insensitive	MSTRG.3253.2		Sensitive (Tetraploid)
Octoploid	Tetraploid	MSTRG.25276.2	PP2C	Sensitive (Tetraploid)	MSTRG.22620.5	HEAT (x2),HEAT_2,Importin_rep_4,Importin_rep_6	Insensitive
Octoploid	Tetraploid	MSTRG.32418.3,MSTRG.32418.4		Insensitive	evm.model.jcf7180004089062.4		Insensitive
Octoploid	Tetraploid	MSTRG.16941.1		Insensitive	evm.model.jcf7180004128298.6	PP2C	Insensitive
Octoploid	Tetraploid	evm.model.jcf7180004134190.2, MSTRG.27161.1		Insensitive	evm.model.jcf7180004108190.4	HATPase_c and HisKA decrease, Exo70 increase	Insensitive
Octoploid	Tetraploid	MSTRG.10338.3	Pribosyltran, POB3_N	Insensitive	MSTRG.33016.1	AMP-binding,AMP-binding_C increase	Sensitive (Octoploid)
Octoploid	Tetraploid	MSTRG.23807.1	Biotin_lipoyl, ACC_central (x2)	Insensitive	evm.model.jcf7180004128736.11	DUF2048 (x2)	Insensitive
Octoploid	Tetraploid	evm.model.jcf7180004094996.3	EF-hand_8 (x2)	Insensitive	MSTRG.4291.3		Insensitive
Octoploid	Tetraploid	evm.model.jcf7180004127787.2	Pollen_Ole_e_1	Insensitive	evm.model.jcf7180004098404.2, MSTRG.10410.1	Methyltransf_2 (one more domain in Tetraploid)	Insensitive
Octoploid	Tetraploid	MSTRG.28064.1,MSTRG.28064.4		Insensitive, Sensitive (MSTRG.28064.4, Octoploid)	evm.model.jcf7180004084359.1, MSTRG.5499.2	PALP	Insensitive
Octoploid	Tetraploid	MSTRG.3765.7, MSTRG.3765.9	WD40	Sensitive (Tetraploid)	MSTRG.23823.1,evm.model.jcf7180004130025.3	Pkinase,PK_Tyr_Ser-Thr	Insensitive
Octoploid	Tetraploid	MSTRG.6510.3	PPR (x9),PPR_2 (x4),PPR_3 (x2)	Sensitive (Octoploid)	evm.model.jcf7180004116421.7, MSTRG.16368.3,MSTRG.16368.4		Insensitive, Sensitive (MSTRG.16368.4, Octoploid)
Octoploid	Tetraploid	MSTRG.34402.4		Sensitive (Octoploid)	MSTRG.16535.2,MSTRG.16535.3		Insensitive, Sensitive (MSTRG.16535.3, Octoploid)
Octoploid	Tetraploid	MSTRG.19828.2		Sensitive (Tetraploid)	MSTRG.29434.1,MSTRG.29434.3	zinc_ribbon_12	Insensitive
Octoploid	Tetraploid	MSTRG.15308.4	RRM_1 (x2)	Insensitive	MSTRG.5773.1	Stress-antifung	Insensitive
Octoploid	Tetraploid	MSTRG.31576.1		Sensitive (Octoploid)	evm.model.jcf7180004139394.1,MSTRG.31634.1	Glycoside hydrolase family	Insensitive
Octoploid	Tetraploid	MSTRG.28737.2	DUF1644	Insensitive	evm.model.jcf7180004083040.4,MSTRG.4813.2		Insensitive
Octoploid	Tetraploid	MSTRG.26492.1	Lactamase_B,Fer4_13	Insensitive	evm.model.jcf7180004097656.2,MSTRG.9836.3	AAA_21,ABC_tran	Insensitive
Octoploid	Tetraploid	MSTRG.26732.2	zf-MYND	Insensitive	evm.model.jcf7180004107336.3		Insensitive
					MSTRG.26324.6,MSTRG.26324.9		Sensitive (Octoploid)
					evm.model.jcf7180004138884.2,MSTRG.31121.2	PMD	Insensitive, Sensitive (MSTRG.31121.2, Octoploid)
					evm.model.jcf7180004135647.3		Insensitive
					evm.model.jcf7180004143412.7,MSTRG.34600.3	SMP (x3),SMP (x2)	Insensitive
					evm.model.jcf7180004090036.1,MSTRG.7576.2,MSTRG.7576.3,MSTRG.7576.4	Sec23_BS,Sec23_helical,Sec23_trunk,zf-Sec23_Sec24	Insensitive, Sensitive (MSTRG.7576.3,MSTRG.7576.4, Octoploid)
					MSTRG.2931.1,MSTRG.2931.2		Sensitive (Tetraploid, Octoploid)
					evm.model.jcf7180004127970.4,MSTRG.21888.2	4F5	Insensitive
					evm.model.jcf7180004098448.2,MSTRG.10464.2		Insensitive
					MSTRG.13123.3		Insensitive
					MSTRG.33255.1,MSTRG.33255.2	Retrotran_gag_2	Insensitive
					MSTRG.32200.1		Sensitive (Tetraploid)
					evm.model.jcf7180004127425.1, MSTRG.21346.2	PHD	Insensitive

### Divergence Time Between Octoploids and Tetraploids

**FIGURE 5:**
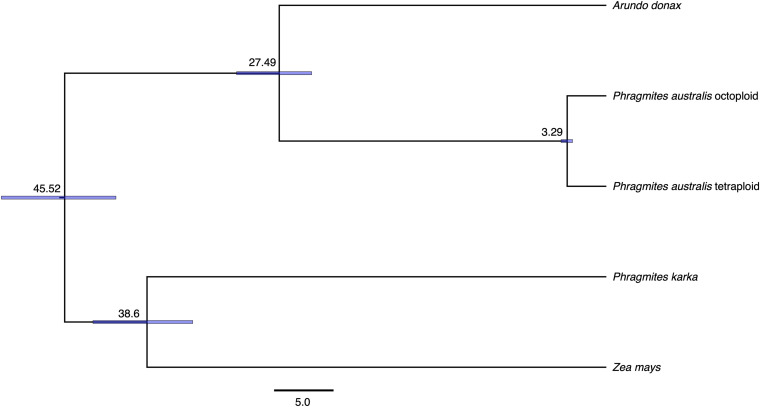
Divergence time estimation of Poaceaespecies based on 98 single copy orthologous genes of five transcriptome assemblies including *Zea mays*, *Arundo donax*, *Phragmites karka*, *Phragmites australis* octoploid lineage, and *Phragmites australis* tetraploid lineage. The unit of the estimated divergence time is million years (MY), and the node bar indicated 95% Height Posterior Density of the node height.

Transcriptome assembly of octoploids contained 180,584 transcripts, with the length of contig N50 being 2,011 bp, and the assembled transcriptome of tetraploids consisted of 167,514 transcripts with the length of contig N50 being 2,046 bp. Orthologue search identified 98 single copy orthologous sequences among the five Poaceae species and dated the divergence time of tetraploid and octoploid lineage of *P. australis* to be 3.26 (95% Highest Posterior Density 2.81–3.69) Mya (Figure 5). Congener species *P. karka* clustered with *Zea mays*, outside of Arundineae, and diverged from Arundineae at 45.36 (95% Highest Posterior Density 41.45–50.75) Mya. *Arundo donax* diverged from *P. australis* at 27.41 (95% Highest Posterior Density 24.63–30.47) Mya. The molecular clock rate was estimated to be 2.17 × 10^–9^ substitutions/year.

## Discussion

### Genome and Differentially Expressed Genes in *Phragmites*

The vast difference between the genomes of higher ploidy levels and lower ploidy levels in plants, has resulted in large gene expression bias, affecting pathways involved in flowering regulation (Braynen et al., 2021) and photosynthetic rate (Ilut et al., 2012). In this study, 1,395 transcripts were found to be differentially expressed between *P. australis* tetraploids and octoploids, and the DEGs were classified into several functional categories which are related to reproduction and resistance to abiotic stresses. DEGs in octoploids were functioning in several pathways, including solute transport (three genes, coding for ABCC transporter, metal cation transporter, or ligand-gated cation channel), protein homeostasis (three genes, coding for cysteine-type peptidase C1 and A1 class of Papain), cytoskeleton organization, RNA processing and polyamine metabolism. Papain-like cysteine proteases are vital enzymes to numerous plant physiological activities, which also function in salt-, cold-, and drought-stress response, as evidenced in model plants such as *Arabidopsis*, wheat, sweet potato, and barley (Liqin et al., 2019). Gene ontology analysis revealed differentially expressed genes upregulated in octoploids enriched in several biological processes, mostly involved in metabolic process, and in error correction mechanisms to environmental stress, such as responses to unfold or incorrectly folded proteins and telomere maintenance. Up to 21 GO terms of these genes were assigned to responses to stress or stimuli, suggesting octoploid *P. australis* has developed many novel functions to cope with the challenging environment. Heat stress may induce abrupt and dramatic loss of telomere DNA repeats (Lee et al., 2016), and genes related to telomere maintenance were upregulated in octoploid *P. australis* to avoid damage to the plant. This is also seen in *Arabidopsis*, where a heat-shock induced molecular chaperone auxiliary maintains the integrity of telomere length under heat stress (Lee et al., 2016). Therefore, we hypothesize that octoploids probably harbor stronger tolerance to heat stress than tetraploids. However, the hypothesis draw from the transcriptomic data may not be conclusive, and more empirical evidence are needed to test the thermotolerance in each ploidy level.

Genes upregulated in tetraploids were involved in cell wall organization through monolignol conjugation and polymerization, and in external stimuli responses in reaction to UV-B light (Table 2). Interestingly, the GO terms were also enriched in biological processes involving development, reproduction processes and seed germination in upregulated genes in tetraploids (Supplementary Table 1), but not in octoploids. Therefore, it seems tetraploids were completing their life cycle faster, whereas octoploids developed more stress tolerance, especially heat resistance which may affect their natural distribution toward warmer territories in lower latitudes. This is further supported in Ren et al. (2020), where two octoploid samples and two tetraploids from this study were caught to flower in year 2017, and it took apparently longer time for the octoploids (266 days) to flower than tetraploids (220 days) (Ren et al., 2020). However, since ploidy information was not included in the study, we cannot draw a solid conclusion on the link between ploidy level and phenology. Therefore, our hypothesis based on transcriptomics data need to be interpreted with caution, and further experiments and developmental characterizations should be introduced to evaluate this theory.

Both of the upregulated and downregulated DEGs annotated in Mercator 4 included multiple genes coding for transcription factors enriched in RNA biosynthesis pathways, genes enriched in phytohormone pathways, cell cycle organization and protein modification. These transcription factors belonging to bZIP, WRKY, MYB, and C2H2 superfamilies, play a crucial role in initiating regulatory networks as response to abiotic stress, such as drought and salinity (Golldack et al., 2014; Han et al., 2020). The sampling process took place in July, the hottest month of the year in Shandong Province, with an average monthly temperature of 32°C during the day. Hence, the hot weather may have constituted a stressful condition for the two groups, and tetraploids and octoploids may have utilized their inherently different pathways to deal with their environment.

Most of the DEGs were enriched in KEGG pathways belonging to “Chaperones and folding catalysts” and “Protein processing in endoplasmic reticulum” (Figure 1B). The heatmap of transcriptome profiles (Figure 2C) showed the most abundant transcripts to be heat shock proteins (HSP70, HSP20), chaperones (HtpG), and ubiquitin C, which are not only essential cellular components that assist with a serial of protein folding processes in cellular compartments, but also modulators of the regulatory network in a crisis of abiotic stress (Usman et al., 2014). For example, high levels of HSP 70 family protein expression have been linked to thermotolerance and resistance to high soil salinity, water stress and high temperature (Wang et al., 2004). UBC gene coding for ubiquitin C is a stress related gene, which correlates positively with higher drought tolerance in *Arabidopsis* and soybean (*Glycine max* (L.) Merr.) by conjugating ubiquitin to remove or unfold damaged proteins (Chen et al., 2020).

### Biased Alternative Isoform Usage May Be Linked to Epigenetic Change

Gene differential expression could be affected by both genetic and epigenetic mechanisms. Liu et al. (2018) pointed out a trend that, as ploidy level increases in *P. australis*, the DNA methylation levels tends to be lower, although this trend was not significant (Liu et al., 2018). DNA methylation in exons or introns at the alternative splicing sites can significantly affect alternative splicing events in gene expression, but not on regularly expressed exons (Shayevitch et al., 2018). In this study, we found that the gene MSTRG.10410 (coding for proteins of the Cation-independent O-methyltransferase family), had a highly expressed isoform (evm.model.jcf7180004098404.2) with two Methyltransf_2 domains in tetraploids, while in octoploids, the gene MSTRG.10410.1 with only one Methyltransf_2 domain was highly expressed (Figure 4A). Methyltransf_2 domain includes a range of O-methyltransferases, which are related to DNA methylation (Keller et al., 1993). Another isoform (evm.model.jcf7180004127425.1) contains one PHD domain in tetraploids, which is responsible for binding to tri-methylated histones or demethylation of proteins, and thus affects the transcription (Schindler et al., 1993). However, the isoform in octoploids (MSTRG.21346.2) is lacking that domain (Figure 4C). These alternate isoforms may have contributed the different methylation level in tetraploids and octoploids. Therefore, the observed alternative splicing events in upregulated and downregulated genes are potentially a reason for, or a result of, the change of DNA methylation levels among ploidy levels. Nonsense-mediated mRNA decay (NMD) identifies cellular mRNAs carrying premature termination codons (PTC), and targets these aberrant transcripts for degradation to prevent the accumulation of potentially deleterious truncated proteins (Shaul, 2015). Moreover, it can also regulate the expression of stress responsive genes in plants, involved in pathways such as pathogen resistance, tolerance to heat shock and temperature change, as well as time-dependent flowering (Staiger and Brown, 2013). The majority of differentially expressed isoforms in *P. australis* were NMD insensitive, and only a few isoforms were NMD sensitive (Table 4). A high proportion of (10 out of 16) NMD sensitive isoforms were biased to be expressed in octoploids, indicating that they are either aberrant transcripts or potentially crucial in defense against environmental stress. In addition, antistress-related isoforms were also found to be differentially expressed between tetraploids and octoploids. For example, isoform MSTRG.5773.1, containing one Stress-antifung domain, was expressed only in tetraploids, although a stronger Stress-antifung (x2) domain was found in octoploids, but this isoform was not expressed significantly higher than in tetraploids (Figure 4B).

### Evolution of Octoploid and Tetraploid Lineages in *Phragmites* Species

The PCA plot separated individuals of the different ploidy levels apart (Figure 1A), suggesting the genetic variation in the samples mainly lies between ploidy level, and not between individuals. Therefore, we included all individuals in each ploidy level and built transcriptome assemblies for tetraploids and octoploids. Based on the PACMAD calibration point, we managed to estimate the divergence time between *A. donax* and *P. australis* at 27.41 Mya, concordant with previous studies which estimated the most common ancestor between *A. donax* and *P. australis* to be 29 Mya (Hardion et al., 2017). *Pragmites karka*, the congener of *P. australis*, clustered with *Zea mays* and diverged from *P. australis* from 45.36 Mya (Figure 5). Ancestors of *Phragmites* have been identified from the Cretaceous Period, so it was not surprising to reveal this divergence between *P. karka* and *P. australis* (Hayden, 1879). Nonetheless, the complicated phylogenetic relationship between the genera of *Phragmites* and *Arundo* suggests that the taxonomic status of Arundineae should be reconsidered.

Divergence between octoploid and tetraploid lineages of *P. australis* was estimated to be 2.81–3.69 Mya, falling at the border between Pliocene and Pleistocene (Bartoli et al., 2011). Pliocene, 5.3–2.6 million years ago, was generally characterized as a warm epoch, with only one mild glaciation cycle described. The onset of Pliocene glaciation started from 3.6 Mya, when the atmospheric CO_2_ decreased transiently until between 3.4 and 3.32 Ma, sea ice volume increased and temperatures cooled down (Bartoli et al., 2011). In mid-Pliocene (3.3–3 Mya), the temperatures rose about 2–3°C higher than in the present atmosphere (Robinson et al., 2008). Pleistocene started from 2.8 Mya, when the warm climate abruptly changed, and intensive glaciation cycles repeatedly occurred. The divergence of octoploid and tetraploid happened shortly after the glaciation at 3.26 Mya, indicating that the two lineages may have experienced bottlenecks and separated in different refugia during glaciation periods which prevented gene flow between lineages, and favored recolonization to new territories during interglacial periods. It has previously been suggested in *Arabidopsis* and Alpine plants that the cool climate, which occurred during glaciation cycles, may have affected cell division during the sensitive period in meiosis, and thereby triggered the generation of polyploids (Sora et al., 2016; Novikova et al., 2018). We cannot draw such a conclusion from our study due to a paucity of information regarding the status of autopolyploidy or allopolyploidy in the investigated tetraploids and octoploids. However, it is worth noticing that there exists more than one lineage of octoploid and tetraploids in *P. australis*. We assign the octoploids to Australian (AU) lineage and tetraploids to European (EU) lineage, based on the geographic locations. Therefore, the estimated divergence time can only date to the most recent common ancestor of *P. australis* AU and EU lineages. Further studies need to be carried out to find the genetic background of different ploidy levels, so as to give a clearer explanation on the evolution of *Phragmites.*

## Data Availability Statement

The datasets generated for this study can be found in the NCBI SRA database with accession number, BioProject PRJNA687616.

## Author Contributions

CW and WG conceived the idea. CW performed data analysis. TW and MY collected the samples and coordinated with sequencing company. FE, LL, and HB maintained the common garden that supplied the samples, CW, WG, FE, TW, MY, LL, and HB led the writing of the manuscript. All authors contributed to the article and approved the submitted version.

## Conflict of Interest

The authors declare that the research was conducted in the absence of any commercial or financial relationships that could be construed as a potential conflict of interest.

## Footnotes

1 http://www.honsunbio.com/

2 https://phytozome.jgi.doe.gov/pz/portal.html, accessed at 2020 November.

3 https://www.arabidopsis.org, accessed at Aug 20th, 2020.

## Supplementary Material

Supplementary Figure 1 Close look to genes contributed to the significant alternative splicing events Exon Skipping/Exon Inclusion (ES/EI). Genes with isoform usage of Exon Skipping (ES) were separated from genes without ES, and comparison between ploidy levels were made to both datasets. Significant isoform usage was indicated with ^∗^. ^∗^*p*-value < 0.05, ^∗∗∗^*p*-value < 0.001, ns, no significant difference.

Supplementary Table 1 Enriched GO terms of DEGs upregulated in tetraploids *P. australis.*

Supplementary Table 2 Enriched GO terms of DEGs upregulated in octoploids *P. australis.*
