# Supplementary figures and images for: Transcriptome Analysis of Tetraploid and Octoploid Common Reed (Phragmites australis)
Source: Front Plant Sci. 2021 May 5;12:653183. doi: 10.3389/fpls.2021.653183 (PMC8132968; doi:10.3389/fpls.2021.653183)

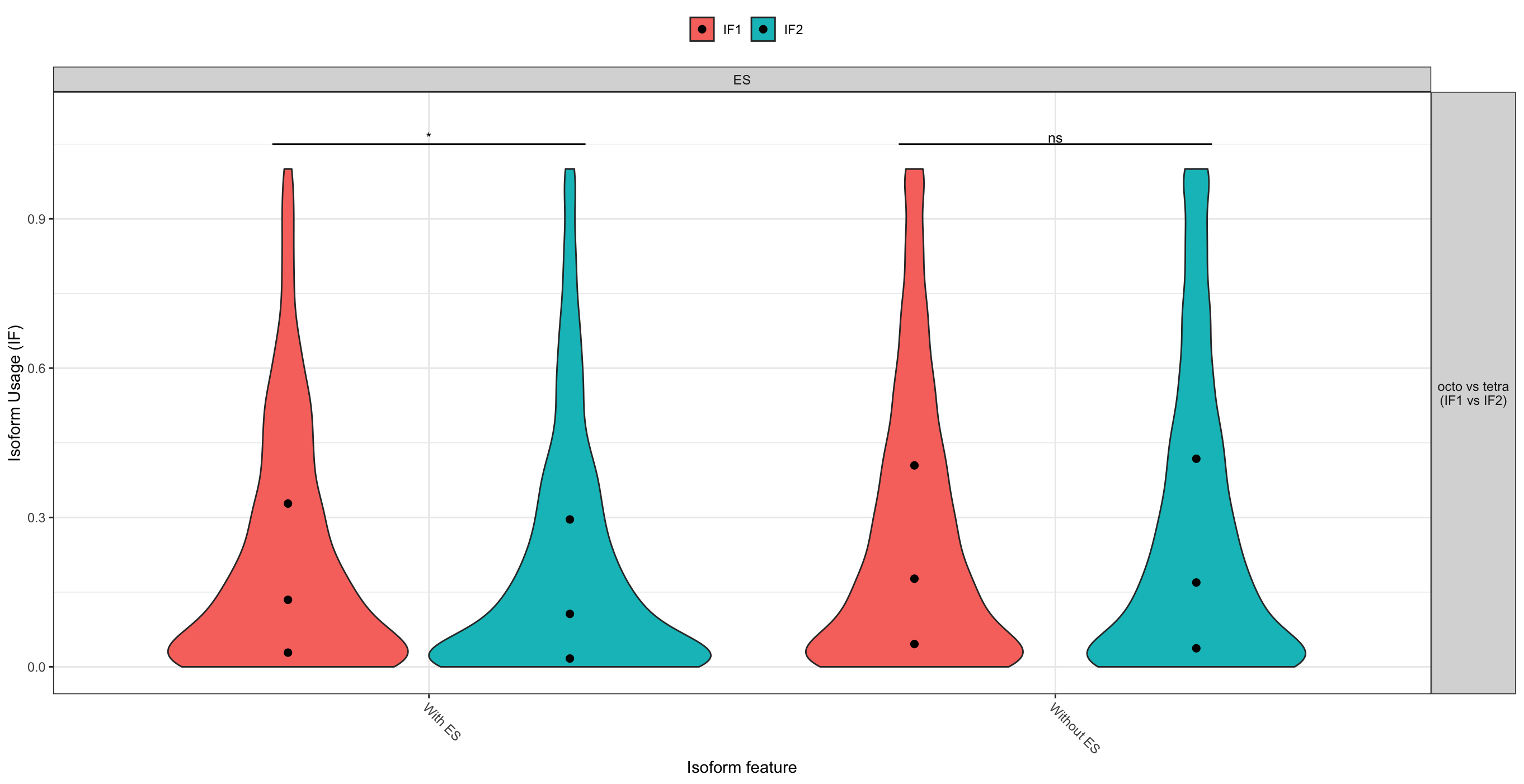

Supplement: Supplementary Figure 1 — Close look to genes contributed to the significant alternative splicing events Exon Skipping/Exon Inclusion (ES/EI). Genes with isoform usage of Exon Skipping (ES) were separated from genes without ES, and comparison between ploidy levels were made to both datasets. Significant isoform usage was indicated with ∗. ∗p-value < 0.05, ∗∗∗p-value < 0.001, ns, no significant difference. [file Image_1.PNG]
